# HIV-related stigma among people living with HIV/AIDS in rural Central China

**DOI:** 10.1186/s12913-018-3245-0

**Published:** 2018-06-15

**Authors:** Zhen Li, Jamie P. Morano, Kaveh Khoshnood, Evelyn Hsieh, Yu Sheng

**Affiliations:** 1 School of Nursing, Peking Union Medical College, Beijing, China; 2 Division of Infectious Diseases and International Medicine, University of South Florida, Tampa, FL USA; 3 Yale University Center for Interdisciplinary Research on AIDS (CIRA), New Haven, USA; 4 Yale School of Public Health, New Haven, CT USA; 5 Department of Internal Medicine, Yale School of Medicine, New Haven, CT USA; 6 Department of Basic Nursing, School of Nursing, Peking Union Medical College, Beijing, China

**Keywords:** Stigma, Human immunodeficiency virus (HIV), Acquired immune deficiency syndrome (AIDS), Plasma donor, Rural

## Abstract

**Background:**

HIV-related stigma among people living with HIV/AIDS (PLWHA) has been associated with many negative consequences, including poor adherence to therapy and undue psychological stress. However, the relative influence of specific demographic and situational factors contributing to HIV-related stigma among rural PLWHA in central China remains unknown. The aim of this study was to explore the level of HIV-related stigma among rural PLWHA across specific demographic and situational factors in central China.

**Methods:**

A cross-sectional study was conducted among PLWHA receiving care through the Chinese Centers for Disease Control of Zhenping county in Henan Province, China. Participants completed a 55-item questionnaire which included demographic and disease-related factors, HIV-related stigma was measured utilizing the validated Berger HIV Stigma Scale which has good psychometric characteristics in Chinese PLWHA.

**Results:**

A total of 239 PLWHA completed the survey. The mean total HIV-related stigma score was 105.92 (SD = 12.35, 95% CI: 104.34, 107.49). Multivariable linear regression analysis revealed a higher level of HIV-related stigma in younger PLWHA (β = − 0.57, 95% CI = − 0.78,-0.35, p<0.001) and those who self-reported opportunistic infections (β = 6.26, 95% CI = 1.26, 11.26, *p* < 0.05).

**Conclusions:**

The findings in the current study suggest that rural PLWHA in central China suffer from the burden of HIV-related stigma at a moderate to high level. Younger PLWHA and PLWHA that have opportunistic infections tend to perceive a higher level of HIV stigma.

## Background

HIV remains a highly stigmatized illness globally [1]. Numerous studies have shown that stigma is associated with poor adherence to antiretroviral therapy (ART) [2], mental health disorders such as loneliness, depression and anxiety [3], non-disclosure of HIV status, and overall poor health outcomes [4, 5]. Because stigma continues to be a major barrier to seeking HIV testing, care, and treatment services [6, 7], it is recognized as a priority for both primary and secondary prevention of HIV and AIDS [8, 9]. Definitions and discussion of different forms of HIV related stigma have been described previously [10].

It is widely accepted that HIV-related stigma is influenced by socio-demographic characteristics such as age, gender, marital status, educational attainment, socioeconomic status, and area of residence. In comparison to urban areas, rural areas are more heavily affected by the HIV epidemic due to difficulty in accessing antiretroviral therapy and logistical challenges in accessing clinical care [11, 12]. In the case of HIV-related stigma, although most attention has been paid to PLWHA living in urban areas, recent studies have demonstrated that PLWHA in rural areas experience a high of level HIV-related stigma [13, 14]. For example, one study in rural Uganda showed that internalized stigma has increased over time among PLWHA [15]. In China, A recent study conducted in individuals from the general population found that the level of stigma towards PLWHA is higher in rural areas than in urban areas [16]. Another study focusing on HIV stigma among older PLWHA from south rural China found that 18.1% reported experiencing external stigma compared with 64.3% experiencing internal stigma [17].

By the end of 2014, there were 510,000 reported cases of people living with HIV/AIDS (PLWHA), and 159,000 deaths had been reported in China [18]. The national HIV/AIDS epidemic maintains a higher-prevalence in areas such as Central China, including the largely agrarian Henan and Hubei provinces. The AIDS epidemic in China first established a rural foothold through illegal, unsanitary commercial plasma donation activities in the late 1980s and early 1990s among unknowing, heterosexual agrarian populations who were paid for plasma donations that involved receiving contaminated pooled blood products [19].

Previous epidemiologic epidemical studies reported that the HIV prevalence among paid plasma donors ranged from 8.6 to 15.1% [20, 21]. This HIV-infected population in rural central China is likely the largest known HIV-infected cohort in the world related to paid plasma donation [22]. This population is also quite different from those in other rural areas in China because they were primarily poor, rural farmers with almost no injection drug use or commercial sex work in their communities [23].

A qualitative study showed that paid blood donors perceived great stigma from the local community [24]. However, limited quantitative data are available regarding the level of HIV related stigma among the population, and it is not clear whether there are specific factors associated with HIV-related stigma. Therefore, in the present study, we measured levels of HIV-related stigma among rural PLWHA in central China with the aim of identifying specific demographic and clinical risk factors associated with higher levels of HIV stigma, so that more targeted and effective anti-stigma strategies can be established for PLWHA in rural China.

## Methods

### Participants

We conducted a cross-sectional and exploratory study in the summer of 2014 at the Centers for Disease Control (CDC) of Zhenping county in Henan province, located in central China. This research site was selected due to its robust HIV/AIDS clinic under the auspices of the China CDC and the large number of HIV positive former plasma donors living in the area.

Inclusion criteria were: (a) documented HIV infection based upon western blot; (b) currently living in an area defined as rural by the local government; (c) > = 18 years of age; and (d) ambulatory. We determined the minimum sample size should be 10–20 times of the number of independent variables. In this study, we evaluated 11 independent factors so we conservatively chose 220 as our sample size. Potentially eligible patients were referred to meet with the study’s trained local research coordinator to determine eligibility, and eligible participants filled out the questionnaire in a private setting. In this study, eligible patients were screened and recruited consecutively during the 2-month study period.

For a few participants (approximately 3% of the sample) with reading difficulties, the research coordinator read items aloud and recorded the participants’ responses on the questionnaire. As a token of gratitude, patients received a toiletry set upon completion of the questionnaire. The study was reviewed and approved by the Research Ethics Committee of Peking Union Medical College prior to initiation.

### Measures

#### Demographics and disease characteristics

All the participants provided demographic and disease status information, including age (years), sex at birth (male, female), current place of residence (rural countryside, rural town), education level (primary school, primary high school or above), marital status (married, unmarried, divorced, or widowed), yearly income (¥CNY), years since HIV diagnosis, history of opportunistic infection by self-report, current antiretroviral therapy (ART), CD4^+^ T-cell count in past three month (cells/mm^3^), self-rated health status (very poor, poor, reasonable, good, very good), and HIV disclosure status.

#### HIV-related stigma

Participants’ HIV-related stigma was measured with the Berger HIV Stigma Scale [25], which is a 40-item tool scored on a four point Likert-type scale; subscales were summed with possible total scores ranging from 40 to 160, with a higher score indicating a higher level of HIV related stigma. Methods for our previous investigation were employed similiarly [10]. The scale included four subscales that were verified by forward and backward translation from English to Mandarin: (1) *personalized stigma* (18 items), which measure the consequences of others knowing about one’s HIV status, including rejection, loss of friends, avoidance of others; (2) *disclosure concerns* (10 items), which measure issues related to whether or not individuals tell others about their diagnosis; (3) *negative self-image* (13 items), which measure one’s feelings towards oneself such as shame, guilt, and self-worth; (4) *concern with public attitudes* (20 items), which measure participants’ perceptions of the public’s attitudes towards those living with HIV/AIDS. Because of the lack of universally accepted cut point of the scores, we adopted the categorization put forward by Charles and colleagues [26], in which the overall stigma scores were categorized into three categories such as no or mild, moderate, and severe stigma using the 33rd and 66th percentile cut off values from the distribution of scores.

### Data analysis

Using PASW SPSS 20.0, descriptive statistics (including frequencies, percentages, mean, or medians with standard deviations) were performed to describe sample characteristics and scores of the Berger HIV Stigma Scale. HIV stigma score percentiles were calculated by dividing the actual subscale scores by the total possible score for that subscale, which made direct comparison of the relative importance of different domains possible. Separate bivariate and multivariable linear regression analyses were used to assess the variables most correlated with HIV stigma. In the regression model, the stigma scores were expressed as a continuous measure. Independent variables included sex at birth (male, female), age, income (<5000CNY (780USD); 5000–10,000CNY (756-1562USD); > 10,000CNY (1562USD)), education level (primary school, primary high school or above), marital status (with spouse, without spouse), years since HIV diagnosis, self-reported opportunistic infection (yes, no), current ART treatment (yes, no), self-rated health status (good or very good, reasonable, poor or very poor), and disclosed HIV status (disclosed, undisclosed). These factors collected and included in the model were determined by our review of the literature. Covariates which were significant in bivariate models with a significance level of < 0.20, were included in the multivariate regression model. A stepwise regression model was established with *p* < 0.05 significance. The data satisfied the assumptions of linear regression testing. No multicollinearity existed among the independent variables.

## Results

### Demographics and disease characteristics

Of 256 individuals screened, a total of 239 agreed to participate and completed the study questionnaire. The mean age of the sample was 51.76 years old (SD = 6.96), ranging from 29 to 74, with 117 (48.95%) men (Table 1). The vast majority of the sample was married (*n* = 191; 79.92%), and had disclosed HIV status to others (*n* = 236; 98.74%). The mean time elapsed since diagnosis was 8.25 years (SD = 3.27), ranging from 1 to 25 years. Those receiving ART made up 97.5% (233/239) of the population, and 72.5% (148/239) had a CD4^+^ T-cell count < 350 cells/ml^3^. A majority (170/2439, 71.1%) rated their health status as reasonable.

**Table 1:** Demographics and disease characteristics of rural people living with HIV/AIDS (PLWHA) in rural Henan Province, China, 2014 (*N* = 239)

Characteristic		n	%
Age (years)			
	≤49	95	39.75
	50–59	105	43.93
	≥60	39	16.32
Gender			
	Male	117	48.95
	Female	122	51.05
Education level			
	Primary school only	166	69.46
	High school or above	73	30.54
Marital status			
	Married	191	79.92
	Unmarried	4	1.67
	Divorced	10	4.18
	Widowed	34	14.23
Yearly income (ҰCNY)			
	≤5000 (780 USD)	79	33.05
	5001–10,000 (756–1562 USD)	147	61.51
	>10,000 (1562 USD)	13	5.44
Years since HIV diagnosis			
	≤9 years	177	74.06
	>9 years	62	25.94
Ever had opportunistic infection (self-reported)			
	Yes	24	10.04
	No	215	89.96
On current ART treatment			
	Yes	233	97.49
	No	6	2.51
CD4+ T-cell count (cells/mm^3^) (*n* = 204)			
	<350 cells/mm^3^	148	72.55
	≥ 350 cells/mm^3^	56	27.45
Self-rated health status			
	Good or very good	18	7.53
	Reasonable	170	71.13
	Poor or very poor	51	21.34
Disclosure of HIV status (to any person)			
	Yes	236	98.74
	No	3	1.26
HIV status disclosed to:			
	Parents (One or both)	88	36.8
	Spouse	128	53.6
	Siblings	87	36.4
	Children	192	80.3
	Health providers	138	57.7
	Close friends	10	4.2
	Relatives	25	10.5

N.B.: *ART* Antiretroviral Therapy, *HIV* Human Immunodeficiency Virus

### Levels of HIV-related stigma

The outcomes of the Berger HIV Stigma Scale with overall mean score and four subscales are shown in Table 2. The overall HIV stigma score ranged from 68 to 130, while the mean score was 105.92 ± 12.35 (95% CI: 104.34, 107.49), and the scores for the four subscales were *personalized stigma* (48.66 ± 6.29), *disclosure concerns* (26.08 ± 3.48), *negative self-image* (34.10 ± 3.99), and *concern with public attitude* (53.47 ± 7.30). According to the percentile of the HIV stigma calculated, the participants perceived greater stigma related to personalized stigma (67.58, 95% CI: 66.47, 68.69) and comparatively lower stigma related to disclosure concerns (65.20, 95% CI: 64.08, 66.30).

**Table 2:** Berger HIV Stigma Scale scores and subscales among rural PLWHA in Henan Province, China, 2014 (N = 239)

Subscales	Number of Items	Average Total Scores	95% CI	Percentile (95% CI)
Personalized Stigma	18	48.66 ± 6.29	47.86, 49.46	67.58 (66.47, 68.69)
Disclosure Concerns	10	26.08 ± 3.48	25.63, 26.52	65.20 (64.08, 66.30)
Negative Self-Image	13	34.10 ± 3.99	33.59, 34.60	65.58 (64.60, 66.54)
Concern with Public Attitude	20	53.47 ± 7.30	52.54, 54.40	66.84 (65.68, 68.00)
Total	40	105.92 ± 12.35	104.34, 107.49	66.20 (65.21, 67.18)

N.B.: *HIV* Human Immunodeficiency Virus, *PLWHA* People Living with HIV/AIDS, *CI* confidence interval

Berger Stigma Score Ranges: personalized stigma (18–72); disclosure concerns (10–40); negative self-image (13–52); concern with public attitude (20–80); Total Score (40–160). These scores are not additive

Values on the Berger HIV Stigma Scale: 0–33% (no or mild stigma); 34–65% (moderate stigma); > 66% (severe stigma)

In addition, a sensitivity analysis was conducted to check for a possible difference between the participants who had reading difficulties (3%) with the remaining participants. We found there was no significant difference in the total stigma score and subscale scores.

### Factors associated with HIV-related stigma

In the bivariate model, it was found that increasing age, increasing education level, living with a spouse and disclosure of HIV status were factors that were significantly associated with *overall* HIV stigma scores.

*Personalized stigma* was significantly correlated with increasing age, increasing education level, current married status, and disclosure of HIV status. *Disclosure concern* was significantly associated with increasing age, increasing education level, and history of opportunistic infection*. Negative self-image* was significantly associated with increasing age, current married status, and history of opportunistic infection. *Concern with public attitudes* was significantly associated with increasing age, increasing education level, married status, and disclosure of HIV status (Table 3).

**Table 3:** Bivariate linear regression models for HIV-related stigma and relevant covariates among rural PLWHA, Henan Province, China, 2014 (*n* = 239)

Variable	Total Stigma Score	Personalized Stigma	Disclosure Concern	Negative Self-Image	Concern With Public Attitudes
	β (95% CI)	β (95% CI)	β (95% CI)	β (95% CI)	β (95% CI)
Female	−1.09 (−4.24,2.07)	− 1.00 (− 2.60,0.60)	− 0.17 (− 1.06,0.72)	−0.28 (− 1.30,0.73)	−0.35 (− 2.22,1.51)
Increasing age	− 0.53 (− 0.75,-0.31)⌘	−0.26 (− 0.37,-0.15)⌘	−0.14 (− 0.20,-0.07)⌘	−0.13 (− 0.20,-0.06)⌘	− 0.30 (− 0.43,-0.17)⌘
Increasing education level	4.40 (1.02,7.78)§	1.93 (0.20,3.66)§	1.82 (0.89,2.76)⌘	0.83 (− 0.27,1.93)*	2.50 (0.50,4.50)§
Living with spouse	5.34 (1.47,9.22)¶	3.02 (1.05,4.99)¶	0.90 (− 0.20,2.01)*	1.69 (0.43,2.94)¶	3.72 (1.44,6.00)⌘
Increasing yearly income	1.35 (− 2.00,4.70)	0.50 (− 1.21,2.20)	0.72 (− 0.22,1.66)*	0.01 (− 1.07,1.09)	0.80 (− 1.18,2.77)
Years since HIV diagnosis	−0.43 (− 0.91,0.05)*	−0.11 (− 0.36,0.14)	−0.13 (− 0.27,0.01)*	−0.15 (− 0.30,0.01)*	−0.27 (− 0.55,0.02)*
Opportunistic infection	4.54 (− 0.68, 9.76)*	1.12 (− 1.55, 3.79)	1.58 (0.12, 3.05)§	1.75 (0.07, 3.42)§	2.07 (− 1.02, 5.16)*
Current ART treatment	1.97 (−8.11,12.04)	2.91(− 2.21,8.04)	−0.25(− 3.09,2.59)	−0.10(− 3.35,3.15)	2.08(− 3.87,8.04)
Better self-rated health status	−1.18 (− 4.22,1.85)	−0.39 (− 1.93,1.16)	−0.32 (− 1.17,0.54)	0.12 (− 0.86,1.10)	−0.67 (− 2.47,1.12)
Disclosed HIV status	−15.61 (− 29.64,-1.59)§	−8.78 (− 15.91,-1.65)§	−2.96 (− 6.94,1.01)*	−3.62 (− 8.16,0.93)*	−9.65 (− 17.93,-1.37)§

*p ≤ 0.2, §*p* < 0.05, ¶*p* < 0.01, ⌘*p* < 0.001

N.B.: A negative stigma score indicates less stigma. *PLWHA* People Living with HIV/AIDS

Opportunistic infection:History of at least one opportunistic infection (self-reported)

In the multivariate model, the *overall* HIV stigma score was negatively associated with increasing age (β = − 0.57, 95% CI:-0.78, − 0.35, *p* < 0.001), and positively associated with self-reported history of opportunistic infection (β = 6.26, 95% CI: 1.26, 11.26, *p* < 0.05) (Table 4).

**Table 4:** Multivariate linear regression models for HIV-related stigma among rural PLWHA, Henan Province, China, 2014 (*n* = 239)

Variable	Total Stigma Score	Personalized Stigma	Disclosure Concern	Negative Self-Image	Concern With Public Attitudes
	β (95% CI)	β (95% CI)	β (95% CI)	β (95% CI)	β (95% CI)
Adjusted R ^2^	0.104	0.093	0.129	0.079	0.105
Female	NA	NA	NA	NA	NA
Increasing age	−0.57(− 0.78, − 0.35)⌘	−0.24(− 0.35, − 0.12)⌘	−0.12(− 0.18,-0.05)⌘	−0.13(− 0.21,-0.06)⌘	−0.28(− 0.42,-0.15)⌘
Increasing education level	–	–	1.25 (0.31,2.19)¶	–	–
Living with spouse	–	2.03 (0.07,4.00)*	–	–	2.54 (0.28,4.81)*
Increasing yearly income	NA	NA	–	NA	NA
Years since HIV diagnosis	–	NA	−0.13(− 0.26,-0.01)*	−0.15(− 0.30,-0.01)*	–
Opportunistic infection	6.26 (1.26, 11.26)*	NA	2.08 (0.68, 3.49)¶	2.37 (0.72, 4.01)¶	2.97(0.01, 5.91)*
Current ART treatment	NA	NA	NA	NA	NA
Better self-rated health status	NA	NA	NA	NA	NA
Disclosed HIV status	–	–	–	–	–
R^2^	0.11	0.10	0.14	0.09	0.12

NA: not included the multivariate model as *p*-value > 0.2 in the univariate model

--: not included the multivariate model as *p*-value >0.05 in the multivariate model

*p < 0.05, ¶*p* < 0.01, ⌘*p* < 0.001

PLWHA: People Living with HIV/AIDS

Opportunistic infection:History of at least one opportunistic infection (self-reported)

In terms of the four subscales, we found that *personalized stigma* was positively correlated with married status and negatively associated with increasing age; *disclosure concern* was positively correlated with increasing education level and history of opportunistic infection, while negatively associated with increasing age and years since HIV diagnosis. *Negative self-image* was positively correlated with history of opportunistic infection and negatively associated with increasing age and years since diagnosis. *Concern with public attitudes* was positively correlated with being married and history opportunistic infection, and negatively associated with increasing age (Table 4).

## Discussion

The present study is one of the most detailed exploring HIV-related stigma among PLWHAs in rural Central China and uncovers important findings. First, we found there is a moderate level of HIV-related stigma existing among the study sample. Secondly, linear regression analysis identified that participants with a younger age, an education level of primary high school or above, currently married status, and history of opportunistic infections were associated with higher HIV-related stigma.

The finding that PLWHA in the rural area of Henan province perceived HIV-related stigma at a moderate to high level is consistent with the literature from China and India [17, 27]. Also, this score is lower than previous study conducted in China among men who have sex with men (MSM) population [10], suggesting that rural former plasma donors are viewed in a more sympathetic light within the general population as compared to those infected with HIV by other routes such as men who have sex with men (MSM), people who inject drugs (PWID), and commercial sex workers (CSW) [28]. Recent studies demonstrate that people who contracted HIV from “blameless” routes (e.g., blood transfusion, sex with stable partners) are less stigmatized compared to people who contracted HIV from “blamable” routes (e.g., injection drug use, homosexual behavior) [29, 30]. Thus, the level of stigma in this rural sample is relatively lower that seen in other higher risk groups. Second, as the sample was recruited from a government-run HIV/AIDS clinic, the study participants may already have received support and resources and developed coping mechanisms that reduced levels of perceived stigma.

Increasing age was associated with lower overall HIV-related stigma scores and the four subscale scores. This is consistent with previous studies among PLWHA in other regions of China [30, 31] and other countries [32, 33]. Emlet and colleagues argued that although encounters with prejudice and discrimination might not lessen with age, it is possible that the overall internalization of those enacted messages may indeed lessen with time [33]. In addition, the older PLWHA may also have more social and family support to adjust and understand the implications of their illness, thus resulting in lower levels of HIV-related stigma.

The presence of opportunistic infections makes it difficult to conceal HIV positive disease status [34] and we found a correlation between history of opportunistic infection and increased HIV stigma scores among our study population suggesting individuals with self-reported history of opportunistic infection bear a higher level of HIV-related stigma than those without. Indeed, Kaai and colleagues found that maintaining good physical health contributes to self-worth and reducing negative public attitudes [35]. HIV stigma-focused interventions continue to be in need of development, both targeting HIV-negative persons and targeting vulnerable groups of PLWHA, such as younger PLWHA and those living with opportunistic infections.

Other factors influencing the four stigma subscales were also identified. A higher education level was positively associated with *disclosure concern*. This is consistent with a study among PLWHA in Finland [36], in which high education level and financial difficulties were associated with greater self-stigma. This result may be explained by that PLWHA with a higher education may be more likely to concern about the damage to social position brought by HIV infection, thus leading to more concern with HIV disclosure [37].

Marital status was an influencing factor of *personalized stigma* and *concern with public attitudes*. Respondents who were currently living with spouses reported experiencing more HIV stigma. This is contrary to previous studies among MSM and older PLWHA in China [17, 38]. A possible reason for the result in the current study may be that only 53.6% (128/239) of the participants disclosed their HIV status to spouses, so for those who are living with spouses, they may face more pressure to keep their HIV status a secret, which results in a higher level of HIV-related stigma.

Years since HIV diagnosis was found to be inversely associated with *disclosure concern* and *negative self-image*. This is consistent with a study among African Americans [39]. With the extension of the time since diagnosis, PLWHA are more likely to receive more information and develop a deeper understanding of the disease and establish psychological mechanisms or seek enough social support to better cope with HIV stigma, resulting in a comparatively lower level of HIV-related stigma.

### Limitations

Our study has certain limitations. First, the fact that data collection was carried in one rural county in central China limits the generalizability of the results to urban populations. Second, the cross-sectional design prevented the exploration of the directionality of the associations between HIV stigma and related factors, so a causal model remains to be tested. Finally, the value of R squared is relatively low for the limited variables included in the model, as other aspects could contribute to HIV related stigma, such as social support and depression. Future studies should address these limitations to advance knowledge on HIV-related stigma.

## Conclusion

Our study provides important implications of HIV-related stigma among an under-studied population of rural PLWHA in central China that has been deeply affected by HIV/AIDS. A deeper understanding of the characteristics of HIV-related stigma among the population is needed to overcome challenges related to access to care and thus to improve overall health outcomes among rural PLWHA. The current study suggests that PLWHA suffer from the burden of HIV-related stigma at a moderate to high level, and those who are younger, and those with opportunistic infections, experience a higher level of HIV-related stigma. Therefore, stigma reduction interventions are needed in these vulnerable groups, and the interventions would benefit from increasing support services for PLWHA.

## Abbreviations

AIDS: Acquired immune deficiency syndrome
ART: Antiretroviral therapy
CDC: Centers for Disease Control
HIV: Human immunodeficiency virus
PDR: People’s Democratic Republic
PLWHA: People living with HIV/AIDS
